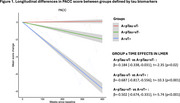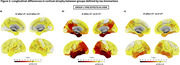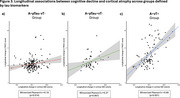# Utility of tau biomarkers for detecting clinical outcomes in preclinical Alzheimer's disease

**DOI:** 10.1002/alz70856_102890

**Published:** 2025-12-25

**Authors:** Miguel A Labrador‐Espinosa, Alexis Moscoso Rial, Nicolai Franzmeier, Michael Schöll

**Affiliations:** ^1^ Department of Psychiatry and Neurochemistry, Institute of Physiology and Neuroscience, University of Gothenburg, Gothenburg, Sweden, Gothenburg, Gothenburg, Sweden; ^2^ Wallenberg Centre for Molecular and Translational Medicine, University of Gothenburg, Gothenburg, Gothenburg, Sweden; ^3^ Department of Psychiatry and Neurochemistry, Institute of Neuroscience and Physiology, The Sahlgrenska Academy, University of Gothenburg, Gothenburg, Sweden; ^4^ Wallenberg Centre for Molecular and Translational Medicine, University of Gothenburg, Gothenburg, Sweden; ^5^ Institute for Stroke and Dementia Research (ISD), LMU University Hospital, LMU, Munich, Bavaria, Germany; ^6^ University of Gothenburg, Gothenburg, Västra Götalands län, Sweden; ^7^ Department of Psychiatry and Neurochemistry, Institute of Physiology and Neuroscience, University of Gothenburg, Gothenburg, Västra Götalands län, Sweden; ^8^ Department of Neuropsychiatry, Sahlgrenska University Hospital, Gothenburg, Gothenburg, Sweden; ^9^ Department of Neurodegenerative Disease, UCL Institute of Neurology, London, United Kingdom

## Abstract

**Background:**

While amyloid‐β pathology is a hallmark of preclinical Alzheimer's disease (AD), tau pathology is a key biomarker for early disease‐modifying interventions due to its strong prognostic association with cortical atrophy and cognitive decline. Plasma *p*‐tau217 offers a cost‐effective measure of tau pathology but lacks regional specificity, reflecting a combination of tau and amyloid‐β pathologies. Alternatively, [18F]flortaucipir PET (tau‐PET) clinical visual‐read provides precise regional insights into advanced tau pathology. This study evaluates the comparative prognostic value of these biomarkers in predicting cognitive decline and neurodegeneration at preclinical AD stages.

**Method:**

We analysed data from 334 cognitively unimpaired participants (mean age: 72.2±4.8 years, 57% female) with amyloid‐β PET positivity (A+:centiloid≥24.4) enrolled in the A4‐LEARN cohort, all of whom had plasma *p*‐tau217Lilly measures and tau‐PET scans at baseline. Plasma *p*‐tau217Lilly positivity (pTau+) was determined using a cut‐off value ≥ 0.28 pg/mL as previously described by the core A4 group *(Sperling, et al. JPrevAlzDis.2024)*, and tau‐PET positivity (vT+) was assessed visually by three specialized readers (Fleiss's κ=0.953 [0.923–0.981]). Based on these biomarkers, participants were classified into A+pTau‐vT‐ (*n* = 185), A+pTau+vT‐ (*n* = 55), and A+vT+ (*n* = 94) groups. Longitudinal changes in PACC scores (median: 12 assessments/subject) and cortical gray matter (GM) volume from serial MRI scans (median: 6 scans/subject) were analyzed over 7.4 years using linear mixed‐effects models. Associations between cognitive decline and cortical atrophy were evaluated by correlating the individual slopes of PACC score decline with those of GM loss.

**Result:**

Compared to the A+pTau‐vT‐ group, significant longitudinal declines in PACC scores were observed in the A+pTau+vT‐ (t=‐2.35, *p* = 0.02) and A+vT+ groups (t=‐10.3, *p* <0.001), with a more pronounced decline in the A+vT+ group (t=‐5.74, *p* <0.001) [Figure 1]. Severe longitudinal GM loss in fronto‐temporo‐parietal regions of the AD‐typical pattern was observed in the A+vT+ group, while only mild effects were noted in the A+pTau+vT‐ group [Figure 2]. Interestingly, the strongest association between cognitive decline and cortical atrophy was observed in the A+vT+ group (r=0.60, *p* <0.001) [Figure 3].

**Conclusion:**

Biomarkers based on plasma *p*‐tau217 levels and visual assessments of tau‐PET demonstrate differential prognostic capabilities, with positive visual tau‐PET reads emerging as a superior predictor of cognitive decline and neurodegeneration, even at preclinical AD stages.